# Combined effects of growth hormone and testosterone replacement treatment in heart failure

**DOI:** 10.1002/ehf2.12520

**Published:** 2019-11-07

**Authors:** Andrea Salzano, Alberto M. Marra, Michele Arcopinto, Roberta D'Assante, Vincenzo Triggiani, Enrico Coscioni, Daniela Pasquali, Giuseppe Rengo, Toru Suzuki, Eduardo Bossone, Antonio Cittadini

**Affiliations:** ^1^ IRCCS SDN Diagnostic and Nuclear Research Institute Naples Italy; ^2^ Department of Translational Medical Sciences, Division of Internal Medicine & Metabolism & Rehabilitation Federico II University Naples Italy; ^3^ Interdisciplinary Department of Medicine, Section of Internal Medicine, Geriatrics, Endocrinology, and Rare Diseases University Aldo Moro Bari Italy; ^4^ Department ‘Cuore’ University Hospital San Giovanni di Dio e Rungi d'Aragona Salerno Italy; ^5^ Department of Neurological, Metabolic, and Geriatric Sciences, Endocrinology Unit University of Campania Luigi Vanvitelli Caserta Italy; ^6^ Istituti Clinici Scientifici Maugeri SpA Società Benefit (ICS Maugeri SpA SB) Telese Terme Benevento Italy; ^7^ Department of Cardiovascular Sciences and NIHR Leicester Biomedical Research Centre University of Leicester Leicester UK; ^8^ Cardiology Division A Cardarelli Hospital Naples Italy; ^9^ Interdisciplinary Research Centre in Biomedical Materials (CRIB) Naples Italy

**Keywords:** Heart failure, Hormones, Growth hormone, Testosterone, Anabolism, Treatment

## Abstract

**Aims:**

Although preliminary studies have demonstrated safety and effectiveness of single replacement therapy for growth hormone deficiency or testosterone deficiency in heart failure (HF), no data are available regarding the combined treatment with both GH and T in this setting. Thus, the aim of the present hypothesis generating pilot study was to evaluate the effectiveness and safety of multiple hormonal replacement therapies in chronic HF.

**Methods and results:**

Five stable HF with reduced ejection fraction patients, with a concomitant diagnosis of growth hormone deficiency and testosterone deficiency, on top of guideline‐based HF treatment underwent 1 year of GH replacement therapy by subcutaneous injections of somatotropin at a dose of 0.012 mg/kg every second day. After 12 months, a T replacement treatment was added at a dosage of 1000 mg every 3 months. Each patient underwent a complete M‐mode, two‐dimensional, and Doppler echocardiographic examination, and an incremental symptom‐limited cardiopulmonary exercise test on a bicycle ergometer at baseline (BL), after 1 year of GH treatment (V1), and after 1 year of combined GH + T treatments (V2). One‐year of GH treatment resulted in a significant improvement in left ventricular ejection fraction (+5.4%, *P* < 0.01), New York Heart Association functional class (*P* < 0.05), and peak oxygen consumption (VO_2_ peak) (+19.3%, *P* < 0.01), and in a significant reduction in NT‐proBNP levels (−35.1%, *P* < 0.01). Notably, one additional year of combined GH and T replacement therapy induced a further increase in VO_2_ peak (+27.7%, final delta change + 52.44%, *P* < 0.01), as well as a significant improvement in muscular strength, as assessed by handgrip dynamometry (+17.5%, final delta change + 25.8%, *P* < 0.01). These beneficial effects were paralleled with an improvement of the overall clinical status (as assessed by New York Heart Association class). Of note, neither adverse effects nor cardiovascular events were reported during the follow‐up period.

**Conclusions:**

Our preliminary data suggest for the first time that combined replacement therapy with GH and T could be considered safe and therapeutic in HF patients with multiple hormone deficiencies, supporting the hypothesis that multiple hormone deficiencies syndrome can be considered as a novel and promising therapeutic target in HF. Further studies with a more robust design and larger population are needed.

## Introduction

Growing evidence suggests that multiple hormone deficiencies (MHD) are common in heart failure (HF) patients and are related to impaired cardiovascular performance and poor outcome.^1^, ^2^, ^3^, ^4^, ^5^, ^6^ Preliminary clinical trials of single hormone replacement therapy to treat growth hormone deficiency (GHD)^7^, ^8^, ^9^ or testosterone deficiency (TD)^10^, ^11^, ^12^ have reported promising results, showing both safety and effectiveness in HF patients.

Notably, although MHD syndrome affects at least one third of the HF population,^2^, ^4^, ^5^ no data are available so far dwelling upon combined GH and T treatment in HF patients. This information is of great relevance because both GH and T are endowed with potential adverse effects.

## Aims

The aim of the current hypothesis generating pilot study was to assess the effects of combined correction of MHD on cardiovascular performance and clinical status in HF patients with reduced ejection fraction. Thus, we have investigated for the first time the effectiveness and safety of combined GH and T replacement therapy in the clinical setting of stable chronic HF.

## Methods

Five stable chronic HF patients with reduced ejection fraction [New York Heart Association (NYHA) Classes II to III], a sub‐group of the control cohort of a previous protocol (NCT01576861),^8^, ^9^ with the diagnosis of both GHD, using the growth hormone releasing hormone plus arginine stimulation test, and TD, according to published guidelines,^13^, ^14^ underwent 1 year of GH replacement therapy to correct GHD by subcutaneous injections of somatotropin (recombinant DNA origin) (Saizen©, Merck Serono International, Geneva, Switzerland) at a dose of 0.012 mg/kg every second day, on top of guideline‐based HF therapy.^15^ After 12 months, because of the presence of signs and symptoms of TD, patients were evaluated eligible to add T replacement treatment (intramuscular testosterone undecanoate, Nebid©, Bayer, Germany) at a dosage of 1000 mg every 3 months. Due to the lack of data regarding safety of combined treatments, in order to minimize potential side effects (e.g. water retention and peripheral oedema) GH and T administration was not started simultaneously. Of note, this strategy allowed us to better characterize additional or synergistic actions of both hormones.

Each patient underwent a complete M‐mode, two‐dimensional, and Doppler echocardiographic examination and an incremental symptom‐limited cardiopulmonary exercise test on a bicycle ergometer at baseline (BL), after 1 year of GH treatment (V1), and after 1 year of combined GH + T treatments (V2). Samples collection and all procedures performed at baseline were repeated annually, while intermediate visits (each 6 months) included clinical assessment and record of clinical events. Patients were treated with evidence‐based therapies (beta‐blockers, ACE/ARBs, and MRA) at a targeted dose^15^ from at least 3 months before the start of hormone deficiencies (HD) replacement therapy in order to minimize possible confounding effects.

Normally distributed continuous variables were expressed as mean ± standard deviation, whereas continuous data with skew distributions were expressed as median and interquartile range. Categorical variables were expressed as counts and percentages. The distribution of the parameters was tested with Kolmogorov–Smirnov test. The intergroup differences were tested with the one‐way ANOVA, with Bonferroni correction as appropriate. Normally distributed parameters were compared between two groups using the *t*‐test paired sample test. Non‐normally distributed parameters were compared between two groups using Mann–Whitney *U*‐test. *P* values <0.05 were deemed statistically significant. All data were analysed using ibm spss Statistics (v24, IBM Corp., Armonk, NY, USA).

## Results

**Figure 1:**
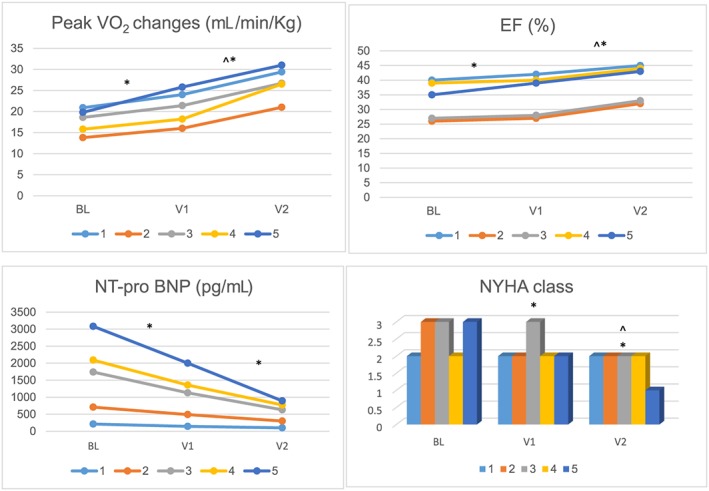
Value at baseline (BL), after 1 year of growth hormone (GH) treatment (V1), and 1 year of GH treatment + testosterone treatment (V2) for selected parameters. EF, ejection fraction; NT‐pro BNP, serum amino terminal fragment of the pro‐hormone brain type natriuretic peptide; NYHA, New York Heart Association; VO_2_,: oxygen consumption. ^*^ *P* < 0.01 respect BL; ^^^ *P* < 0.01 V2 respect V1.

Baseline characteristics of enrolled patients are depicted in *Table* 1. One‐year of GH treatment resulted in a significant increase in left ventricular (LV) ejection fraction (EF) of 5.4% and in VO_2_ peak of 19.3% (approximately 1.8% and 3.4 mL/min/kg, respectively), paralleled by a non‐significant trend in decreasing VE/VCO_2_ slope (about 7.6% corresponding to 2.2 mL/min/kg) (*Table* 2). Consistently, a statistically significant reduction in NT‐pro BNP levels of 35% and a significant improvement in NYHA functional class were observed. A small increase in HOMA‐IR was detected (although not statistically significant). No relevant effects were observed with regard to body mass index and handgrip performance. With the addition of T replacement therapy for an additional year, cardiopulmonary and skeletal muscle performance improved, while no effect was detected on LV structure. Notably, EF significantly increased of an additional 12% (final delta change: +18%, *P* < 0.01), while VO_2_ peak significantly increased by an average of 5.84 mL/min/kg, (+ 27.7%, final delta change from baseline 52.4%, *P* < 0.01). A 17.5% improvement in handgrip performance was equally observed (final delta change of 25.8%, *P* < 0.01). All these results were paralleled by a striking improvement in NYHA class and a reduction in NT‐pro BNP levels (*P* < 0.01) (*Figure* 1). As expected, total testosterone and sex hormone binding globulin levels, which remained substantially stable during the first phase of the study, changed following T treatment. In particular, total testosterone increased, whereas sex hormone binding globulin decreased (+132.9% and −40.1%, respectively), resulting in a significant increase in free T and bioavailable T (+261.5% and +308.3%, respectively). HF treatment dosages (all patients were treated with evidence‐based therapies—beta‐blockers, ACE/ARBs, and MRA—at a targeted dose from at least 3 months before baseline) remained stable (expressed as >75% of the targeted dose) during the study period, as well as non‐pharmacological approaches (e.g. diet). No patients received anticoagulant drugs. Adverse effects were reported neither during GH alone (e.g. fluid retention, hypertension, paraesthesia, joint stiffness, peripheral oedema, arthralgia, myalgia, and carpal tunnel syndrome) nor during combined therapy (GH + T). Following T treatment, no significant changes were observed with regard to prostatic specific antigen and haematocrit. One patient during GH treatment and one patient during the GH + T treatment had a self‐limiting flu‐like syndrome, which did not require any medical intervention and were not related to drug administration. Finally, no major adverse cardiovascular events occurred during the study period.

**Table 1:** Detailed characteristics of each patient at time of enrolment

ID patients	1	2	3	4	5
Age (year)	62	55	70	67	48
BMI (kg/m^2^)	30	28	31	25	36
NYHA class	2	3	3	2	3
EF (%)	40	26	27	39	35
Peak VO_2_ (mL/min/kg)	20.9	13.8	18.6	15.8	19.2
VE/VCO_2_ slope	25.8	34.6	22.5	28.1	32.4
NT‐pro BNP (pg/mL)	211	497	1031	352	992
ESVi (mL/m^2^)	174	179	178	140	187
EDVi (mL/m^2^)	291	242	244	229	288
Handgrip (kg)	31	33	21	27	43
Glycaemia (mg/dL)	87	98	92	85	79
Insulin (microU/mL)	17.15	5.05	3.38	4.11	14.5
HOMA IR index	3.68	1.22	0.77	0.86	2.82
IGF‐1 (ng/mL)	51	63	46	30	133
Total Testosterone (ng/dL)	202	190	187	142	144
SHBG (nmol/L)	36.2	57.9	24.2	43.4	36.5
Free testosterone (ng/dL)	3.60	2.34	3.40	1.52	2.39
Bioavailable testosterone (ng/dL)	84.5	58.8	97.8	52	60.4

BMI, body mass index; EDV, end diastolic volume; EF, ejection fraction; ESV, end systolic volume; HOMA‐IR, homeostasis model assessment‐ insulin resistance; IGF‐1, insulin growth factor‐1; NT‐pro BNP, serum amino terminal fragment of the pro‐hormone brain type natriuretic peptide; NYHA, New York Heart Association; VCO_2_, carbon dioxide production; VE, ventilation per minute; VO_2_, oxygen consumption.

**Table 2:** Value at baseline (BL), after 1 year of growth hormone (GH) treatment (V1), and 1 year of GH treatment + testosterone treatment (V2); delta (Δ) change after 1 year of GH treatment (ΔV1), after 1 year of GH treatment + testosterone treatment compared from previous time point (ΔV2) and overall effect (ΔEP)

Characteristics	Values			ANOVA		Delta changes		
	Baseline	V1	V2	*f*	*P*	ΔV1	ΔV2	ΔEP
BMI (kg/m^2^)	30 ± 4	29.8 ± 3.6	30.4 ± 3.8	—	—	−0.2 (−0.7%)	0.6 (2%)	0.4 (1.3%)
NYHA class (I/II/III)	0/2/3	0/4/1*	1/4/0* ^,^ §	3.7	0.05	−0.4 (15.4%)	−0.4 (18.2%)	−0.8 (30.8%)
EF (%)	33.4 ± 6.6	35.3 ± 7.12*	39.4 ± 6.34* ^,^ §	156	<0.001	1.8 (5.4%)	4.2 (11.9%)	6 (17.9%)
Peak VO_2_ (mL/kg/min)	17.6 ± 2.8	21.1 ± 4.0*	26.9 ± 3.8* ^,^ §	24.5	<0.001	3.4 (19.3%)	5.84 (27.7%)	9.26 (52.44)
VE/VCO_2_ slope	28.7 ± 4.9	26.5 ± 4.4	24.8 ± 4.2	—	—	−2.2 (−7.6%)	−1.7 (−6.4%)	−3.9 (−13.5%)
NT‐pro BNP (pg/mL)	497 [352–1031]	347 [228–643]*	142 [120–330]*	8.5	0.01	−216 (−35.1%)	−221.6 (−55%)	−438.2 (−71.1%)
ESVi (mL/m^2^)	171.6 ± 18.3	159.8 ± 13	145.4 ± 11.5	—	—	−11.8 (6.9%)	−14.4 (−9%)	−26.2 (−15.3%)
EDVi (mL/m^2^)	258.8 ± 28.6	249.4 ± 26.7	242 ± 25.8	—	—	−9.4 (3.6%)	−7.4 (−2.9%)	−16.8 (−6.5%)
Handgrip	31 ± 8.1	33.2 ± 7.7	39 ± 8.2* ^,^ §	323	< 0.001	2.2 (7.1%)	5.8 (17.5%)	8 (25.8%)
Glycaemia (mg/dL)	88.2 ± 7.19	90.4 ± 2.97	87.2 ± 3.27	—	—	2.2 (2.5%)	−3.2 (−3.5%)	−1 (−1.14)
Insulin (microU/mL)	8.83 ± 6.47	9.96 ± 6.23	9.65 ± 6.07	—	—	1.12 (12.7%)	−0.3 (−3.1%)	0.82 (9.2%)
HOMA‐IR index	1.9 ± 1.3	2.2 ± 1.3	2.1 ± 1.3	—	—	0.34 (18.8%)	−0.15 (−6.78)	0.19 (10.2%)
IGF‐1 (ng/mL)	51 [46–133]	113 [100–158]*	146 [135–201]*	63	<0.001	45.5 (70.9%)	33.4 (30.3%)	79.2 (122.6%)
Testosterone (ng/dL)	187 [144–202]	179 [161–204]	389 [351–488]* ^,^ §	126	<0.001	−1 (−0.6%)	228.6 (132.9%)	227.6 (131.6%)
SHBG (nmol/L)	36.5 [36.2–57.9]	35.8 [35–59.3]	24.6 [17.5–33.1]* ^,^ §	3.98	0.05	0.7 (1.8%)	−16.2 (−40.1%)	−15.5 (−39.1%)
Free testosterone (ng/dL)	2.58 [2.51–4.17]	2.39 [2.34–3.63]	9.19 [7.8–12]* ^,^ §	23	<0.001	−0.36 (−11.9%)	6.9 (261.5%)	6.58 (218.3%)
Bioavailable testosterone (ng/dL)	60.4 [58.8–97.8]	54.9 [52.5–88.9]	230 [187–304]* ^,^ §	36.9	<0.001	−13.1 (−18.5%)	177.6 (308.3%)	164.5 (232.7%)

Data are expressed as mean ± standard deviation or median (interquartile range).

* *P* < 0.05 respect BL.

§ *P* < 0.05 V2 respect V1.

BMI, body mass index; EDV: end diastolic volume; EF: Ejection fraction; ESV: End systolic volume; HOMA‐IR: homeostasis model assessment‐insulin resistance; IGF‐1: insulin growth factor‐1; NT‐pro BNP: serum amino terminal fragment of the pro‐hormone brain type natriuretic peptide; NYHA: New York Heart Association; VCO_2_: carbon dioxide production; VE: ventilation per minute; VO_2_: oxygen consumption.

## Conclusions

The current hypothesis generating pilot study suggests that combined hormone replacement therapy yields overall beneficial effects on a wide array of cardiovascular parameters. Consistently with previous findings related to GH or T treatment,^16^, ^17^, ^18^, ^19^, ^20^ in our population, GH appears to improve mostly LV architecture and function (as mainly demonstrated by the EF improvement),^9^, ^16^, ^17^ while testosterone therapy results in ameliorated skeletal muscle performance, possibly due to peripheral vasodilation and improvement in oxygen delivery to skeletal muscle, and slightly reduction in insulin resistance.^12^, ^18^, ^21^ Of note, after the 2 years follow‐up, no drug‐related adverse effects were observed suggesting that both GH and T treatment are safe, even if combined, as previously showed in healthy individuals.^22^, ^23^, ^24^ Finally, no major adverse cardiovascular events occurred during the study.

The present finding is in line with the notion that HD can be considered as a novel, safe, and promising therapeutic target in HF.^25^, ^26^ In this regard, the American Heart Association recommends to test for GHD in patients with dilated cardiomyopathy who have signs and symptoms of GHD,^27^ and the eventual presence of the primary deficit in GH levels should be appropriately treated (namely, GH cardiomyopathy).^27^ Most recent European guidelines considered T therapy as a possible treatment for cachexia and sarcopenia in combination with nutritional supplements.^15^

There are several limitations of the current study. First, it involved only a small group of patients enrolled from a single centre. Second, the lack of a placebo arm. Further, it has not been designed to provide mechanistic explanation of the observed phenomena. Finally, it is not possible to clearly discriminate the effects of the two treatments on each different variable. All these limitations are related to the design of the protocol, and further investigations in more robust randomized clinical trial studies are needed. However, the strength of the present report is to be the first that investigated the effects of combined GHD and TD replacement therapy in HF. Thus, these promising preliminary results could be viewed as a background for the implementation of more robust clinical trials.

In conclusion, despite several limitations, the data from this hypothesis generating pilot study support the idea that the combination of GH and T replacement therapy in the treatment of concomitant HD seems to have beneficial effect on the cardiovascular performance in HF patients. However, further studies on larger populations and with a more robust study design are needed.

## Conflict of interest

None declared.
